# Synthesis and structure of a 1:1 1-phenyl­semicarbazide–1-phenyl­pyrazolidin-3-one cocrystal

**DOI:** 10.1107/S2056989026002550

**Published:** 2026-03-17

**Authors:** S. Muthu, V. L. Siji, S. S. Sreejith, M. Sithambaresan

**Affiliations:** a Department of Chemistry, Sree Narayana College, Varkala, India; b Department of Chemistry, Mahatma Gandhi College, Thiruvananthapuram, India; c Department of Chemistry, All Saints’ College, Thiruvananthapuram, India; d Department of Chemical Oceanography Lake Side Campus Cochin University of Science & Technology,Kochi India; e Department of Chemistry Faculty of Science Eastern University, Sri Lanka, Chenkalady Sri Lanka; University of Aberdeen, United Kingdom

**Keywords:** crystal structure, cocrystal, *gauche* conformation

## Abstract

The title cocrystal, C_7_H_9_N_3_O·C_9_H_10_N_2_O, was obtained by the cocrystallization of 1-phenyl­semicarbazide (*A*) and 1-phenyl­pyrazolidin-3-one (*B*) in a 1:1 molar ratio from methanol solution. The structure features a *gauche* arrangement about the N—N bond in the semicarbazide fragment and a twisted conformation of the pyrazolidinone ring. In the extended structure, the mol­ecules are linked by *A* → *A* and *B* → *A* N—H⋯O hydrogen bonds, supplemented by C—H⋯π contacts to generate a three-dimensional supra­molecular framework.

## Abstract 2

The title cocrystal was obtained by the cocrystallization of 1-phenyl­semicarbazide (*A*) and 1-phenyl­pyrazolidin-3-one (*B*) in a 1:1 molar ratio from methanol solution. The structure features a *gauche* arrangement about the N—N bond in the semicarbazide fragment and a twisted conformation of the pyrazolidinone ring. In the extended structure, the mol­ecules are linked by *A* → *A* and *B* → *A* N—H⋯O hydrogen bonds, supplemented by C—H⋯π contacts to generate a three-dimensional supra­molecular framework.

## 1. Chemical context

Semicarbazide (CH_5_N_3_O) is the di­amino derivative of urea and contains three N atoms and a carbonyl group as potential hydrogen-bonding sites and for coordination with metal centers (Asifa *et al.*, 2021 ▸). It forms semicarbazones on condensation with aldehydes/ketone (Reena *et al.*, 2008 ▸; Reena & Kurup, 2010 ▸). Consequently, semicarbazide and its derivatives have found extensive utility in the synthesis of compounds with biological and industrial relevance. Beyond its biological applications, semicarbazide derivatives have also been reported to act as effective corrosion inhibitors in aqueous environments (Olasunkanmi *et al.*, 2020 ▸).

In the field of crystal engineering, cocrystals defined as single-phase crystalline materials comprising two or more neutral mol­ecular species have long been exploited to tune physicochemical attributes while preserving the mol­ecular identity of active components (Taylor & Day, 2018 ▸). A recent review reaffirms that cocrystallization remains a powerful approach to modulate API properties, with modern design strategies expanding *via* techniques such as spray drying, hot-melt extrusion and supercritical fluid processing (Sakhiya & Borkhataria 2024 ▸). Rode and colleagues demonstrated the potential of flavonoid-based cocrystal and coamorphous systems (Rode *et al.*, 2024 ▸).
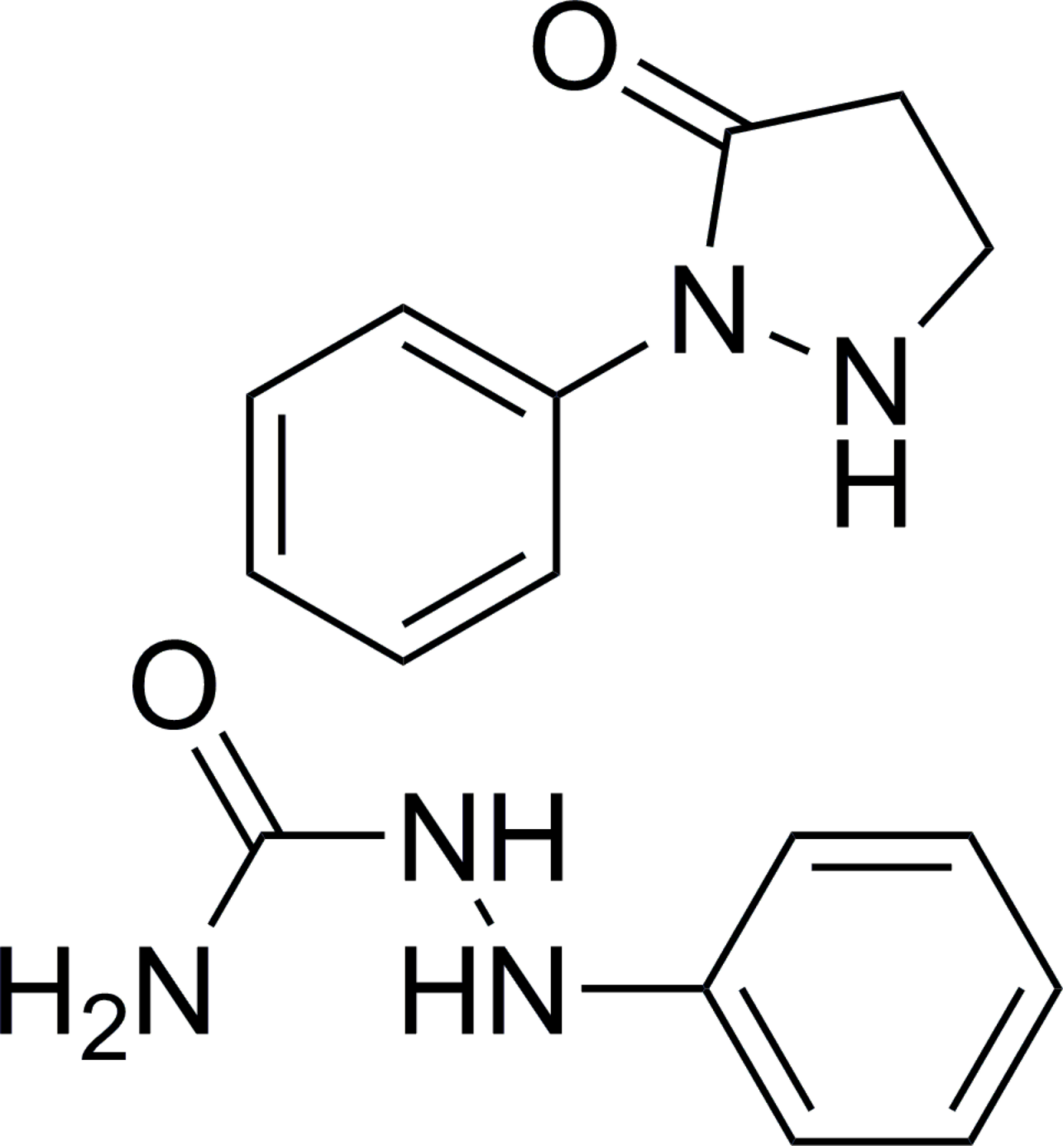


**Figure 1:**
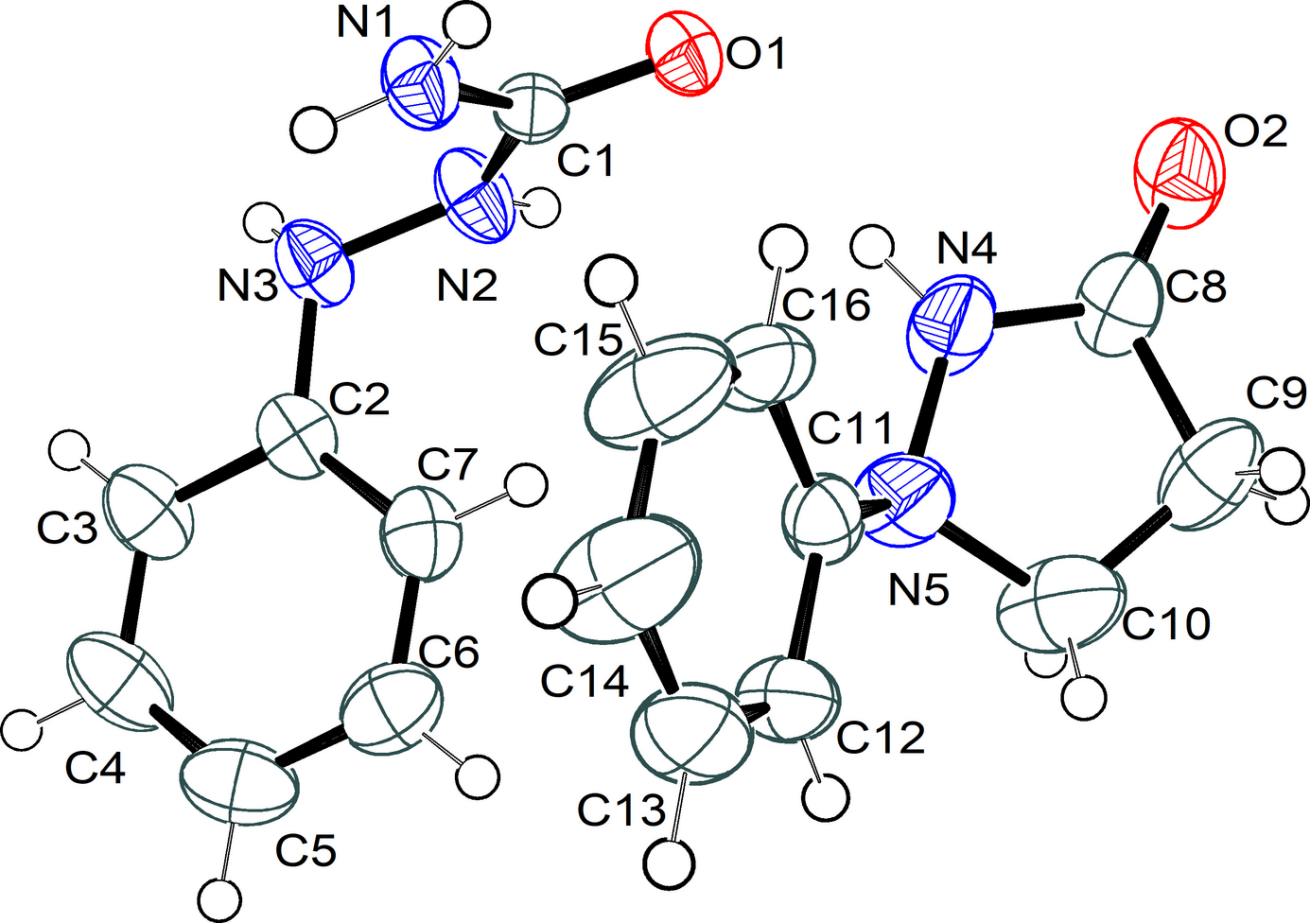
The mol­ecular structure of (**I**) with displacement ellipsoids drawn at the 50% probability level.

As part of our studies in this area, we now describe the synthesis and structure of the title 1:1 cocrystal formed between 1-phenyl­semicarbazide (*A*) and 1-phenyl­pyrazolidin-3-one (*B*), C_7_H_9_N_3_O·C_9_H_10_N_2_O, (**I**), Fig. 1 ▸.

## 2. Structural commentary

The semicarbazide–aromatic linkage in mol­ecule *A* of (**I**) displays a pronounced deviation from coplanarity, as shown by the C1—N2—N3—C2 torsion angle of −99.2 (2)°, indicating a *gauche* arrangement about the N—N bond. The O1—C1—N2—N3 torsion of 177.2 (1)° confirms an anti-periplanar relationship between the carbonyl oxygen atom and the N—N linkage, while the N1—C1—N2—N3 torsion angle of −1.9 (2)° supports near planarity at C1 and *sp^2^* hybridization at N1. In the side chain, C3—C2—N3—N2 [−167.0 (1)°] is close to anti-periplanar, whereas C7—C2—N3—N2 [17.9 (2)°] indicates a *gauche* disposition of the phenyl substituent.

The pyrazolidinone ring (C8–C10/N4/N5) in mol­ecule *B* adopts a twisted conformation about C10—C9, with Cremer–Pople parameters *Q*(2) = 0.229 (2) Å and Φ(2) = 263.7 (5)°. The pseudorotation parameters *P* = 65.5 (4)° and τ_M_ = 23.9 (2)° (Jia *et al.*, 2008 ▸) confirm this geometry. The mean absolute torsion angle (17.6°) and atomic deviations in the ring (–0.142 to +0.132 Å) indicate moderate non-planarity, in line with reported pyrazolidinone structures (Domenicano *et al.*, 1975 ▸). The pyrazolidinone and phenyl (C11–C16) rings are inclined by 54.42 (12)°, producing a significant out-of-plane arrangement.

## 3. Supra­molecular features

The extended structure of (**I**) features an extensive network of inter­molecular inter­actions, including N—H⋯O hydrogen bonds (Table 1 ▸) and C—H⋯π contacts, which collectively underpin its efficient packing and structural cohesion.

**Figure 2:**
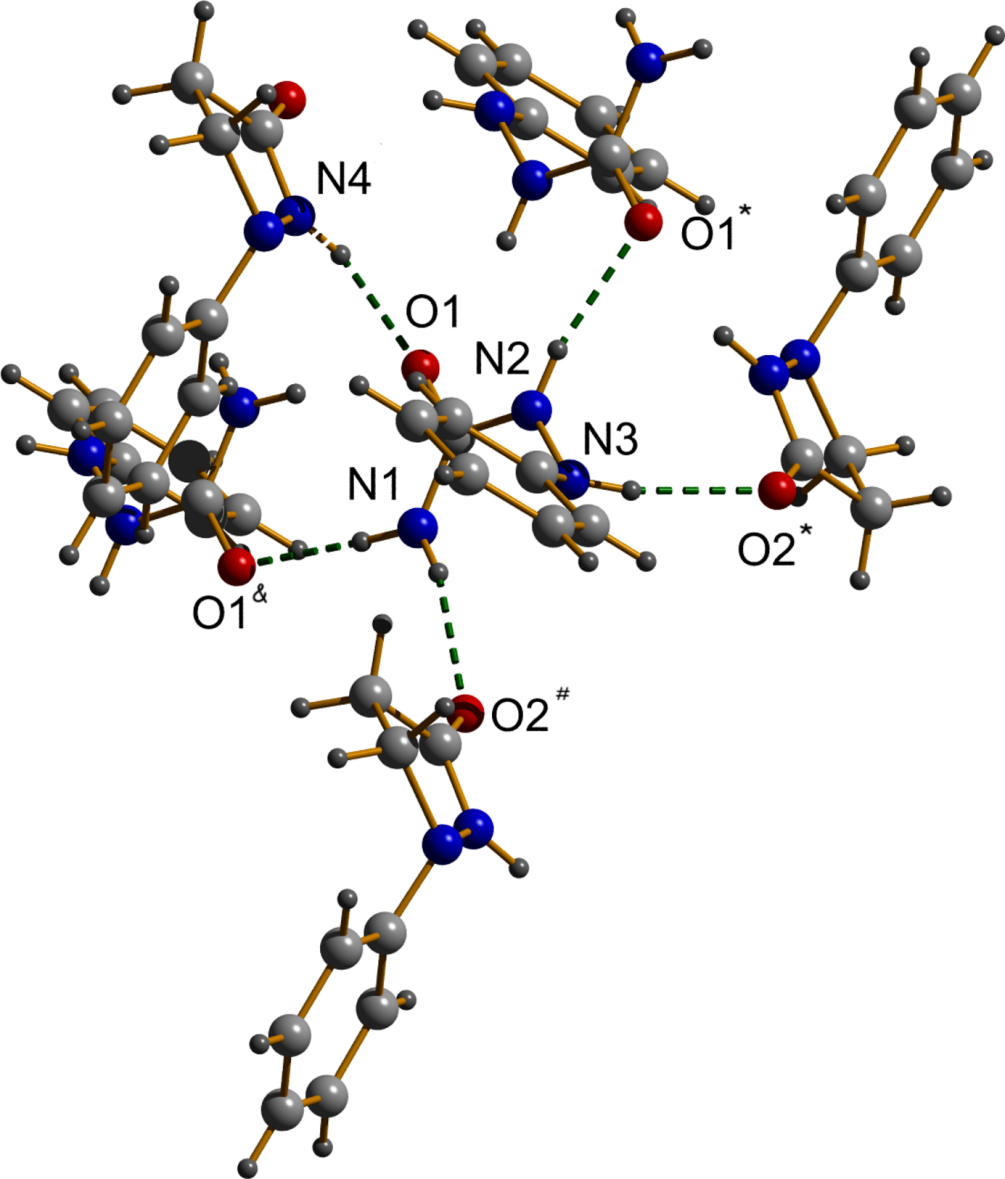
Hydrogen bonds in the crystal packing of (**I**) shown as dashed lines.

Five significant hydrogen bonds were identified, featuring donor–acceptor (*D*⋯*A*) distances shorter than 3.05 Å. These hydrogen bonds all involve N—H donors and carbonyl oxygen atom acceptors (Fig. 2 ▸): the strong, near-linear inter­actions such as N1—H1*B*⋯O1 and N4—H4*N*⋯O1 are consistent with hydrogen-bonding motifs typical of semicarbazide-based structures, contributing to a well-defined supra­molecular framework (Kurup *et al.*, 2011 ▸; Kunnath *et al.*, 2026 ▸).

**Figure 3:**
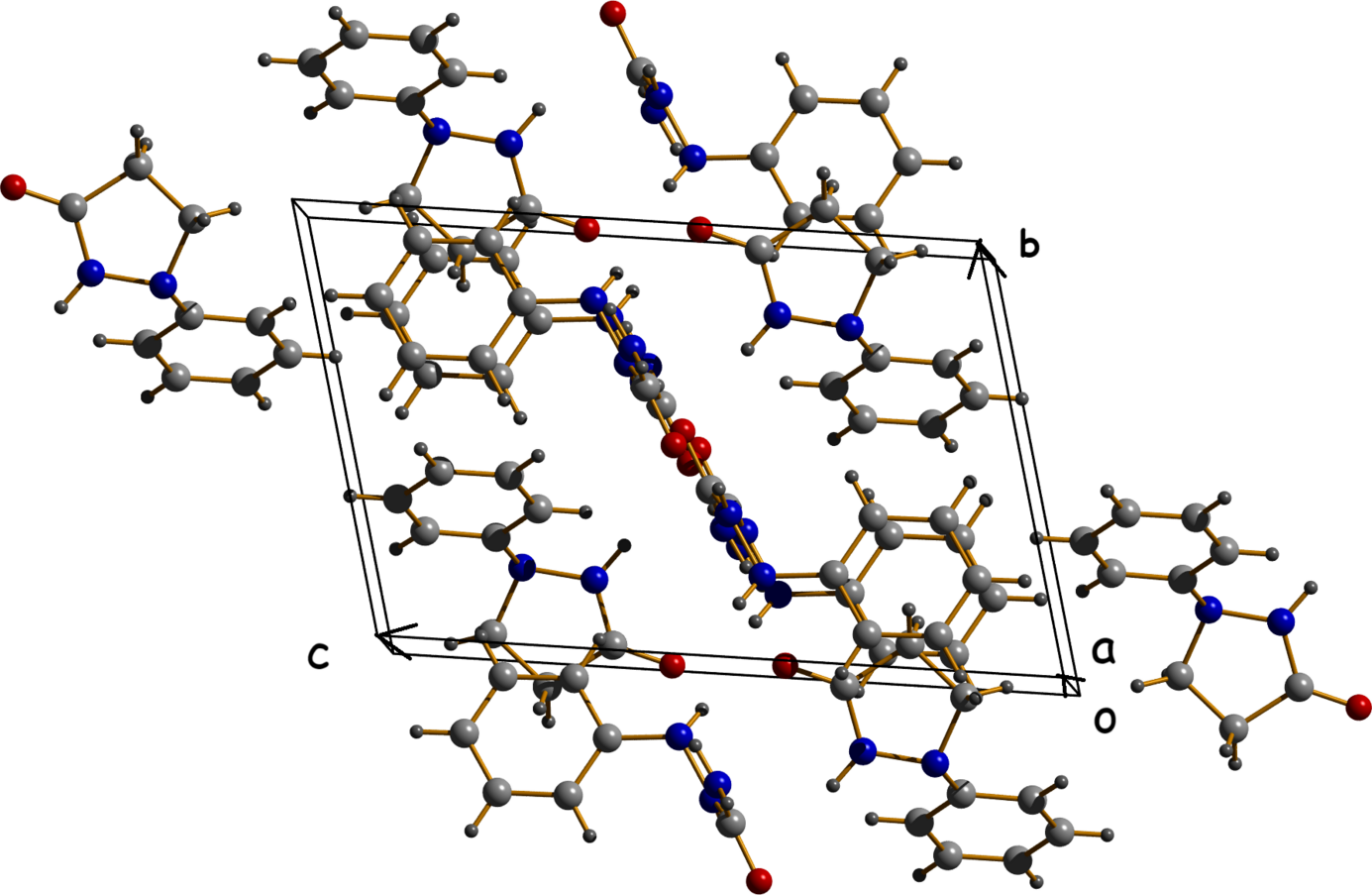
The packing of (**I**) viewed down [100].

Complementing these, C—H⋯π inter­actions consolidate the structure: these inter­actions were identified by short hydrogen-to-centroid distances (< 3.0 Å) and favorable angular geometries (Desiraju & Steiner, 1999 ▸). Two notable C—H⋯π contacts include C4—H4⋯*Cg*2 with an H⋯*Cg* distance of 2.77 Å and C9—H9*B*⋯*Cg*3 with a shorter H⋯*Cg* distance of 2.64 Å. The latter exhibits a more linear approach, facilitating an effective perpendicular hydrogen-bonding inter­action to the π-system (Mahadevi & Sastry, 2016 ▸). These C—H⋯π contacts further enhance the cohesion of the structure of (**I**) but there are no significant π–π inter­actions present in the crystal (Fig. 3 ▸).

## 4. Hirshfeld surface analysis

Hirshfeld surface analysis and the associated two-dimensional fingerprint plots were generated using *CrystalExplorer* (Spackman *et al.*, 2021 ▸). The Hirshfeld surfaces were mapped over the normalized contact distance (*d*_norm_), shape index, curvedness, and fragment patches for both the 1-phenyl­semicarbazide and 1-phenyl­pyrazolidin-3-one components (see supplementary Figure). The *d*_norm_ surfaces of both mol­ecules exhibit bright-red spots that signify close inter­molecular contacts (shorter than the sum of van der Waals radii). For the 1-phenyl­semicarbazide moiety, these red regions are concentrated around the amine donors (N1, N2) and the carbonyl acceptor (O1). Similarly, the 1-phenyl­pyrazolidin-3-one surface shows deep-red depressions near the N4 donor and O2 acceptor atoms. These features confirm the presence of the strong N—H⋯O hydrogen bonding network identified in the crystal structure analysis.

**Figure 4:**
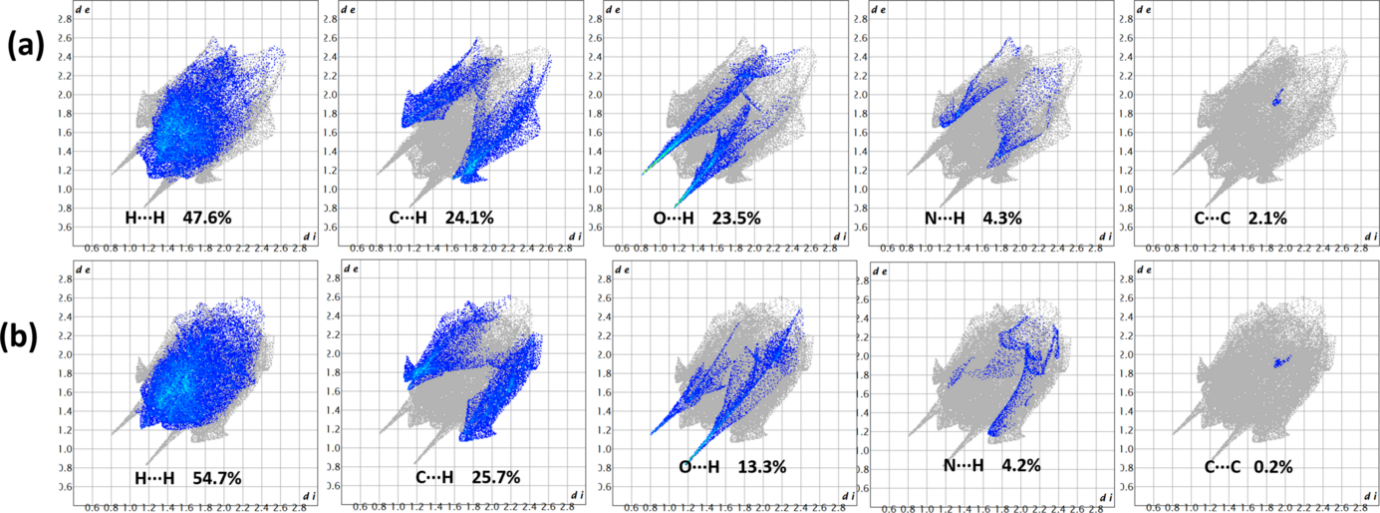
Two-dimensional fingerprint plots for (*a*) 1-phenyl­semicarbazide and (*b*) 1-phenyl­pyrazolidin-3-one, delineated into specific inter­molecular contacts with their percentage contributions to the Hirshfeld surface area.

The two-dimensional fingerprint plots (Fig. 4 ▸) provide a qu­anti­tative comparison of the inter­molecular inter­actions for the two distinct units. The surface of *A* is dominated by H⋯H contacts (47.6%), followed by C⋯H/H⋯C contacts (24.1%), which correspond to the C—H⋯π inter­actions. The O⋯H/H⋯O inter­actions, representing the strong hydrogen bonds, make a significant contribution of 23.5% and appear as sharp, distinct spikes. In *B*, a higher dominance of H⋯H contacts (54.7%) can be seen and a similar contribution from C⋯H/H⋯C inter­actions (25.7%). However, the O⋯H/H⋯O inter­actions contribute less (13.3%) compared to the semicarbazide unit.

## 5. Synthesis and crystallization

Compound (**I**) was formed by mixing equimolar qu­anti­ties of 1-phenyl­semicarbazide (0.1512 g, 1 mmol) and 1-phenyl­pyrazolidin-3-one (0.1621 g, 1 mmol) in methanol (20 ml) and refluxing with stirring until a clear solution was obtained. The hot solution was filtered to remove any insoluble material and allowed to cool slowly to room temperature. Brown block-shaped crystals of (**I**) were obtained after slow evaporation of the solvent over several days.

FT IR (cm^−1^) 3291 (N—H stretch); 3190 (aromatic C—H stretch); 1693 (amine N—H stretch); 1663 (C=O stretch); 1427 (pyrazolidinone C=N stretch); 1282 (aromatic C=N stretch); 1024 (semicarbazide N—N stretch). The red shift for the C=O bond from its typical position substanti­ates the hydrogen bonding in the cocrystal (Siji *et al.*, 2010*a* ▸,*b* ▸). UV/visible (methanol), 245 and 287 nm due to π–π* transitions of the aromatic rings. For figures of the IR, UV/visible and ^1^H and ^13^C NMR spectra of (**I**), see supporting information.

## 6. Refinement

Crystal data, data collection and structure refinement details are summarized in Table 2 ▸. The N-bound hydrogen atoms were located in difference maps and freely refined. The C-bound H atoms were placed geometrically and refined using a riding model.

## Supplementary Material

Crystal structure: contains datablock(s) I. DOI: 10.1107/S2056989026002550/hb8196sup1.cif

Structure factors: contains datablock(s) I. DOI: 10.1107/S2056989026002550/hb8196Isup3.hkl

Additional Figures. DOI: 10.1107/S2056989026002550/hb8196sup4.docx

Supporting information file. DOI: 10.1107/S2056989026002550/hb8196Isup4.cml

CCDC reference: 2378160

Additional supporting information:  crystallographic information; 3D view; checkCIF report

## Figures and Tables

**Table 1:** Hydrogen-bond geometry (Å, °) *Cg*2 and *Cg*3 are the centroids of the C11–C16 and C2–C7 rings, respectively

*D*—H⋯*A*	*D*—H	H⋯*A*	*D*⋯*A*	*D*—H⋯*A*
N1—H1*B*⋯O1^i^	0.93 (2)	2.09 (2)	3.0164 (17)	178 (2)
N1—H1*A*⋯O2^ii^	0.87 (2)	2.12 (2)	2.9645 (18)	161 (2)
N2—H2*N*⋯O1^iii^	0.88 (2)	2.09 (2)	2.9486 (17)	166 (2)
N3—H3*N*⋯O2^iii^	0.84 (2)	2.15 (2)	2.9641 (19)	161 (2)
N4—H4*N*⋯O1	0.88 (2)	2.08 (2)	2.9471 (19)	167 (2)
C4—H4⋯*Cg*2^iv^	0.93	2.77	3.653 (3)	158
C9—H9*B*⋯*Cg*3^v^	0.97	2.64	3.510 (2)	149

Symmetry codes: (i) 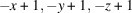; (ii) 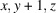; (iii) 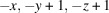; (iv) 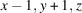; (v) 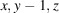.

**Table 2:** Experimental details

Crystal data	
Chemical formula	C_7_H_9_N_3_O·C_9_H_10_N_2_O
*M*_r_	313.36
Crystal system, space group	Triclinic, *P* 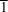
Temperature (K)	304
*a*, *b*, *c* (Å)	7.3651 (6), 8.8906 (5), 13.3791 (10)
α, β, γ (°)	76.121 (2), 88.579 (3), 73.108 (2)
*V* (Å^3^)	812.84 (10)
*Z*	2
Radiation type	Mo *K*α
μ (mm^−1^)	0.09
Crystal size (mm)	0.35 × 0.21 × 0.09

Data collection	
Diffractometer	Bruker D8 Venture Diffractometer
Absorption correction	Multi-scan (*SADABS*; Krause *et al.*, 2015 ▸)
*T*_min_, *T*_max_	0.535, 0.745
No. of measured, independent and observed [*I* > 2σ(*I*)] reflections	32158, 3069, 2566
*R*_int_	0.063
(sin θ/λ)_max_ (Å^−1^)	0.609

Refinement	
*R*[*F*^2^ > 2σ(*F*^2^)], *wR*(*F*^2^), *S*	0.045, 0.123, 1.06
No. of reflections	3063
No. of parameters	223
H-atom treatment	H atoms treated by a mixture of independent and constrained refinement
Δρ_max_, Δρ_min_ (e Å^−3^)	0.22, −0.26

Computer programs: *APEX4* and *SAINT* (Bruker, 2021 ▸), *SHELXT2018/2* (Sheldrick, 2015*a* ▸), *SHELXL2019/3* (Sheldrick, 2015*b* ▸), *ORTEP-3 for Windows* (Farrugia, 2012 ▸), *Mercury* (Macrae *et al.*, 2020 ▸) and *publCIF* (Westrip, 2010 ▸).

## supplementary crystallographic information

### 1-Phenylsemicarbazide–1-phenylpyrazolidin-3-one (1/1) Crystal data

**Table:**

C_7_H_9_N_3_O·C_9_H_10_N_2_O	*Z* = 2
*M**_r_* = 313.36	*F*(000) = 332
Triclinic, *P*1	*D*_x_ = 1.280 Mg m^−^^3^
*a* = 7.3651 (6) Å	Mo *K*α radiation, λ = 0.71073 Å
*b* = 8.8906 (5) Å	Cell parameters from 9264 reflections
*c* = 13.3791 (10) Å	θ = 2.6–25.6°
α = 76.121 (2)°	µ = 0.09 mm^−^^1^
β = 88.579 (3)°	*T* = 304 K
γ = 73.108 (2)°	Block, brown
*V* = 812.84 (10) Å^3^	0.35 × 0.21 × 0.09 mm

### 1-Phenylsemicarbazide–1-phenylpyrazolidin-3-one (1/1) Data collection

**Table:**

Bruker D8 Venture Diffractometer	2566 reflections with *I* > 2σ(*I*)
Radiation source: fine focus sealed tube	*R*_int_ = 0.063
φ and ω scans	θ_max_ = 25.7°, θ_min_ = 2.9°
Absorption correction: multi-scan (SADABS; Krause *et al.*, 2015)	*h* = −8→8
*T*_min_ = 0.535, *T*_max_ = 0.745	*k* = −10→10
32158 measured reflections	*l* = −16→16
3069 independent reflections	

### 1-Phenylsemicarbazide–1-phenylpyrazolidin-3-one (1/1) Refinement

**Table:**

Refinement on *F*^2^	Primary atom site location: structure-invariant direct methods
Least-squares matrix: full	Hydrogen site location: mixed
*R*[*F*^2^ > 2σ(*F*^2^)] = 0.045	H atoms treated by a mixture of independent and constrained refinement
*wR*(*F*^2^) = 0.123	*w* = 1/[σ^2^(*F*_o_^2^) + (0.0602*P*)^2^ + 0.2037*P*] where *P* = (*F*_o_^2^ + 2*F*_c_^2^)/3
*S* = 1.06	(Δ/σ)_max_ < 0.001
3063 reflections	Δρ_max_ = 0.22 e Å^−^^3^
223 parameters	Δρ_min_ = −0.26 e Å^−^^3^
0 restraints	

### 1-Phenylsemicarbazide–1-phenylpyrazolidin-3-one (1/1) Special details

**Table:**

Geometry. All esds (except the esd in the dihedral angle between two l.s. planes) are estimated using the full covariance matrix. The cell esds are taken into account individually in the estimation of esds in distances, angles and torsion angles; correlations between esds in cell parameters are only used when they are defined by crystal symmetry. An approximate (isotropic) treatment of cell esds is used for estimating esds involving l.s. planes.

### 1-Phenylsemicarbazide–1-phenylpyrazolidin-3-one (1/1) Fractional atomic coordinates and isotropic or equivalent isotropic displacement parameters (Å^2^)

**Table:**

	*x*	*y*	*z*	*U*_iso_*/*U*_eq_	
C1	0.2118 (2)	0.60592 (16)	0.52900 (10)	0.0311 (3)	
C2	−0.0439 (2)	0.79617 (18)	0.69126 (12)	0.0372 (3)	
C3	−0.1379 (2)	0.9337 (2)	0.72492 (14)	0.0498 (4)	
H3	−0.197869	1.029765	0.677228	0.060*	
C4	−0.1425 (2)	0.9279 (3)	0.82926 (16)	0.0589 (5)	
H4	−0.206428	1.020272	0.851105	0.071*	
C5	−0.0538 (3)	0.7872 (3)	0.90105 (15)	0.0577 (5)	
H5	−0.056367	0.784348	0.971004	0.069*	
C6	0.0385 (3)	0.6513 (2)	0.86799 (15)	0.0544 (5)	
H6	0.098617	0.555851	0.916145	0.065*	
C7	0.0435 (2)	0.6540 (2)	0.76410 (14)	0.0455 (4)	
H7	0.105433	0.560428	0.743041	0.055*	
C8	0.3195 (2)	0.0381 (2)	0.66850 (14)	0.0480 (4)	
C9	0.3529 (3)	−0.0688 (2)	0.77620 (15)	0.0605 (5)	
H9A	0.486672	−0.126278	0.791363	0.073*	
H9B	0.281541	−0.146969	0.785807	0.073*	
C10	0.2827 (4)	0.0488 (3)	0.84304 (16)	0.0755 (7)	
H10A	0.152873	0.054819	0.861230	0.091*	
H10B	0.362110	0.016182	0.905826	0.091*	
C11	0.4384 (2)	0.26709 (17)	0.80707 (12)	0.0382 (4)	
C12	0.4607 (3)	0.2703 (2)	0.90964 (13)	0.0470 (4)	
H12	0.385872	0.227717	0.958919	0.056*	
C13	0.5924 (3)	0.3359 (2)	0.93827 (15)	0.0617 (5)	
H13	0.608361	0.335093	1.007139	0.074*	
C14	0.7001 (4)	0.4023 (3)	0.86646 (17)	0.0829 (8)	
H14	0.788846	0.446994	0.886201	0.100*	
C15	0.6767 (3)	0.4026 (3)	0.76518 (17)	0.0762 (7)	
H15	0.748700	0.449360	0.716113	0.091*	
C16	0.5478 (3)	0.3346 (2)	0.73500 (13)	0.0502 (4)	
H16	0.534571	0.334117	0.666125	0.060*	
N1	0.3338 (2)	0.68854 (16)	0.53556 (11)	0.0399 (3)	
N2	0.02993 (19)	0.66632 (17)	0.55207 (11)	0.0439 (3)	
N3	−0.02852 (19)	0.80919 (16)	0.58501 (11)	0.0420 (3)	
N4	0.2967 (3)	0.18810 (18)	0.67682 (12)	0.0585 (5)	
N5	0.2939 (2)	0.20624 (18)	0.77994 (11)	0.0529 (4)	
O1	0.25943 (14)	0.47328 (12)	0.50354 (8)	0.0368 (3)	
O2	0.3102 (2)	−0.00009 (15)	0.58679 (10)	0.0601 (4)	
H1B	0.460 (3)	0.641 (3)	0.5246 (17)	0.072*	
H1A	0.303 (3)	0.777 (3)	0.5578 (17)	0.072*	
H2N	−0.052 (3)	0.614 (3)	0.5465 (17)	0.072*	
H3N	−0.115 (3)	0.879 (3)	0.5449 (18)	0.072*	
H4N	0.267 (3)	0.273 (3)	0.6235 (18)	0.072*	

### 1-Phenylsemicarbazide–1-phenylpyrazolidin-3-one (1/1) Atomic displacement parameters (Å^2^)

**Table:**

	*U*^11^	*U*^22^	*U*^33^	*U*^12^	*U*^13^	*U*^23^
C1	0.0351 (8)	0.0324 (7)	0.0255 (7)	−0.0095 (6)	−0.0015 (5)	−0.0065 (5)
C2	0.0246 (7)	0.0426 (8)	0.0471 (9)	−0.0090 (6)	0.0004 (6)	−0.0171 (7)
C3	0.0372 (9)	0.0512 (10)	0.0570 (11)	0.0018 (7)	−0.0051 (7)	−0.0229 (8)
C4	0.0397 (9)	0.0741 (13)	0.0656 (12)	−0.0020 (9)	0.0045 (8)	−0.0403 (10)
C5	0.0451 (10)	0.0854 (14)	0.0469 (10)	−0.0208 (9)	0.0080 (8)	−0.0224 (10)
C6	0.0515 (10)	0.0601 (11)	0.0512 (11)	−0.0231 (9)	0.0024 (8)	−0.0044 (8)
C7	0.0422 (9)	0.0412 (9)	0.0555 (10)	−0.0134 (7)	0.0038 (7)	−0.0148 (7)
C8	0.0496 (10)	0.0431 (9)	0.0544 (10)	−0.0201 (7)	−0.0144 (8)	−0.0077 (7)
C9	0.0653 (12)	0.0539 (11)	0.0631 (12)	−0.0344 (9)	−0.0157 (9)	0.0068 (9)
C10	0.1078 (18)	0.0908 (16)	0.0520 (12)	−0.0705 (15)	0.0022 (11)	−0.0117 (11)
C11	0.0393 (8)	0.0336 (7)	0.0391 (8)	−0.0066 (6)	−0.0051 (6)	−0.0085 (6)
C12	0.0520 (10)	0.0540 (10)	0.0369 (9)	−0.0181 (8)	0.0031 (7)	−0.0119 (7)
C13	0.0833 (14)	0.0691 (12)	0.0412 (10)	−0.0337 (11)	−0.0108 (9)	−0.0142 (9)
C14	0.1059 (19)	0.1016 (18)	0.0610 (13)	−0.0729 (16)	−0.0196 (12)	−0.0022 (12)
C15	0.0874 (16)	0.0977 (17)	0.0542 (12)	−0.0617 (14)	−0.0031 (11)	0.0043 (11)
C16	0.0622 (11)	0.0532 (10)	0.0330 (8)	−0.0192 (8)	−0.0039 (8)	−0.0027 (7)
N1	0.0368 (7)	0.0382 (7)	0.0507 (8)	−0.0140 (6)	0.0053 (6)	−0.0184 (6)
N2	0.0330 (7)	0.0478 (8)	0.0601 (9)	−0.0121 (6)	0.0026 (6)	−0.0299 (7)
N3	0.0386 (7)	0.0398 (7)	0.0458 (8)	−0.0013 (6)	−0.0046 (6)	−0.0188 (6)
N4	0.0910 (12)	0.0433 (8)	0.0427 (8)	−0.0255 (8)	−0.0237 (8)	−0.0035 (6)
N5	0.0604 (9)	0.0597 (9)	0.0448 (8)	−0.0272 (7)	−0.0093 (7)	−0.0117 (7)
O1	0.0369 (6)	0.0357 (5)	0.0416 (6)	−0.0114 (4)	0.0025 (4)	−0.0157 (4)
O2	0.0730 (9)	0.0490 (7)	0.0625 (8)	−0.0192 (6)	−0.0141 (7)	−0.0182 (6)

### 1-Phenylsemicarbazide–1-phenylpyrazolidin-3-one (1/1) Geometric parameters (Å, º)

**Table:**

C1—O1	1.2554 (16)	C10—H10A	0.9700
C1—N1	1.3311 (19)	C10—H10B	0.9700
C1—N2	1.3489 (19)	C11—C16	1.380 (2)
C2—C7	1.389 (2)	C11—C12	1.394 (2)
C2—C3	1.391 (2)	C11—N5	1.416 (2)
C2—N3	1.403 (2)	C12—C13	1.372 (3)
C3—C4	1.384 (3)	C12—H12	0.9300
C3—H3	0.9300	C13—C14	1.365 (3)
C4—C5	1.376 (3)	C13—H13	0.9300
C4—H4	0.9300	C14—C15	1.369 (3)
C5—C6	1.372 (3)	C14—H14	0.9300
C5—H5	0.9300	C15—C16	1.379 (3)
C6—C7	1.384 (3)	C15—H15	0.9300
C6—H6	0.9300	C16—H16	0.9300
C7—H7	0.9300	N1—H1B	0.93 (2)
C8—O2	1.229 (2)	N1—H1A	0.87 (2)
C8—N4	1.326 (2)	N2—N3	1.3909 (18)
C8—C9	1.505 (3)	N2—H2N	0.88 (2)
C9—C10	1.508 (3)	N3—H3N	0.84 (2)
C9—H9A	0.9700	N4—N5	1.426 (2)
C9—H9B	0.9700	N4—H4N	0.88 (2)
C10—N5	1.475 (2)		
			
O1—C1—N1	122.83 (13)	H10A—C10—H10B	108.8
O1—C1—N2	118.62 (13)	C16—C11—C12	118.72 (15)
N1—C1—N2	118.54 (13)	C16—C11—N5	122.61 (15)
C7—C2—C3	118.78 (15)	C12—C11—N5	118.47 (15)
C7—C2—N3	122.29 (14)	C13—C12—C11	120.35 (17)
C3—C2—N3	118.76 (15)	C13—C12—H12	119.8
C4—C3—C2	120.15 (17)	C11—C12—H12	119.8
C4—C3—H3	119.9	C14—C13—C12	120.53 (18)
C2—C3—H3	119.9	C14—C13—H13	119.7
C5—C4—C3	120.84 (17)	C12—C13—H13	119.7
C5—C4—H4	119.6	C13—C14—C15	119.57 (19)
C3—C4—H4	119.6	C13—C14—H14	120.2
C6—C5—C4	119.08 (17)	C15—C14—H14	120.2
C6—C5—H5	120.5	C14—C15—C16	120.87 (19)
C4—C5—H5	120.5	C14—C15—H15	119.6
C5—C6—C7	121.05 (17)	C16—C15—H15	119.6
C5—C6—H6	119.5	C15—C16—C11	119.93 (17)
C7—C6—H6	119.5	C15—C16—H16	120.0
C6—C7—C2	120.10 (16)	C11—C16—H16	120.0
C6—C7—H7	119.9	C1—N1—H1B	117.0 (13)
C2—C7—H7	119.9	C1—N1—H1A	122.8 (14)
O2—C8—N4	124.66 (16)	H1B—N1—H1A	120 (2)
O2—C8—C9	128.52 (16)	C1—N2—N3	120.99 (13)
N4—C8—C9	106.80 (16)	C1—N2—H2N	119.1 (14)
C8—C9—C10	103.49 (16)	N3—N2—H2N	119.9 (15)
C8—C9—H9A	111.1	N2—N3—C2	117.65 (13)
C10—C9—H9A	111.1	N2—N3—H3N	110.9 (15)
C8—C9—H9B	111.1	C2—N3—H3N	118.1 (15)
C10—C9—H9B	111.1	C8—N4—N5	114.89 (15)
H9A—C9—H9B	109.0	C8—N4—H4N	122.9 (14)
N5—C10—C9	105.18 (16)	N5—N4—H4N	121.5 (14)
N5—C10—H10A	110.7	C11—N5—N4	114.65 (14)
C9—C10—H10A	110.7	C11—N5—C10	117.83 (15)
N5—C10—H10B	110.7	N4—N5—C10	104.08 (14)
C9—C10—H10B	110.7		
			
C7—C2—C3—C4	0.4 (2)	C12—C11—C16—C15	0.3 (3)
N3—C2—C3—C4	−174.90 (16)	N5—C11—C16—C15	175.19 (18)
C2—C3—C4—C5	0.4 (3)	O1—C1—N2—N3	177.20 (13)
C3—C4—C5—C6	−0.6 (3)	N1—C1—N2—N3	−1.9 (2)
C4—C5—C6—C7	0.1 (3)	C1—N2—N3—C2	−99.21 (17)
C5—C6—C7—C2	0.7 (3)	C7—C2—N3—N2	17.9 (2)
C3—C2—C7—C6	−0.9 (2)	C3—C2—N3—N2	−166.97 (14)
N3—C2—C7—C6	174.18 (14)	O2—C8—N4—N5	173.73 (17)
O2—C8—C9—C10	−160.94 (19)	C9—C8—N4—N5	−4.9 (2)
N4—C8—C9—C10	17.7 (2)	C16—C11—N5—N4	12.7 (2)
C8—C9—C10—N5	−23.4 (2)	C12—C11—N5—N4	−172.37 (14)
C16—C11—C12—C13	−1.6 (3)	C16—C11—N5—C10	135.77 (19)
N5—C11—C12—C13	−176.65 (16)	C12—C11—N5—C10	−49.3 (2)
C11—C12—C13—C14	1.6 (3)	C8—N4—N5—C11	119.86 (17)
C12—C13—C14—C15	−0.3 (4)	C8—N4—N5—C10	−10.3 (2)
C13—C14—C15—C16	−1.0 (4)	C9—C10—N5—C11	−107.49 (18)
C14—C15—C16—C11	1.0 (4)	C9—C10—N5—N4	20.7 (2)

### 1-Phenylsemicarbazide–1-phenylpyrazolidin-3-one (1/1) Hydrogen-bond geometry (Å, º)

*Cg*2 and *Cg*3 are the centroids of the C11–C16 and C2–C7 rings, respectively

**Table:**

*D*—H···*A*	*D*—H	H···*A*	*D*···*A*	*D*—H···*A*
N1—H1*B*···O1^i^	0.93 (2)	2.09 (2)	3.0164 (17)	178 (2)
N1—H1*A*···O2^ii^	0.87 (2)	2.12 (2)	2.9645 (18)	161 (2)
N2—H2*N*···O1^iii^	0.88 (2)	2.09 (2)	2.9486 (17)	166 (2)
N3—H3*N*···O2^iii^	0.84 (2)	2.15 (2)	2.9641 (19)	161 (2)
N4—H4*N*···O1	0.88 (2)	2.08 (2)	2.9471 (19)	167 (2)
C4—H4···*Cg*2^iv^	0.93	2.77	3.653 (3)	158
C9—H9*B*···*Cg*3^v^	0.97	2.64	3.510 (2)	149

Symmetry codes: (i) −*x*+1, −*y*+1, −*z*+1; (ii) *x*, *y*+1, *z*; (iii) −*x*, −*y*+1, −*z*+1; (iv) *x*−1, *y*+1, *z*; (v) *x*, *y*−1, *z*.

## References

[bb1] Asifa, S., Khan, M. S., Ahmad, S. & Husain, F. M. (2021). *J. Mol. Struct.* **1249**, 131442.

[bb2] Bruker (2021). *SAINT* and *APEX4*. Bruker AXS Inc., Madison, Wisconsin, USA.

[bb3] Desiraju, G. R. & Steiner, T. (1999). *The Weak Hydrogen Bond in Structural Chemistry and Biology*. Oxford University Press.

[bb4] Domenicano, A., Vaciago, A. & Coulson, C. A. (1975). *Acta Cryst.* B**31**, 1630–1641.

[bb5] Farrugia, L. J. (2012). *J. Appl. Cryst.* **45**, 849–854.

[bb6] Jia, H.-S., Li, Y.-F., Liu, Y.-Y., Liu, S. & Zhu, H.-J. (2008). *Acta Cryst.* E**64**, o855. 10.1107/S1600536808009823 PMC2961278 21202342

[bb7] Krause, L., Herbst-Irmer, R., Sheldrick, G. M. & Stalke, D. (2015). *J. Appl. Cryst.* **48**, 3–10. 10.1107/S1600576714022985 PMC4453166 26089746

[bb8] Kunnath, R. J., Seena, E. B., Sithambaresan, M., Patel, M., Jeong, B., Raju, M. & Kurup, M. R. P. (2026). *Polyhedron* **289**, 118002.

[bb9] Kurup, M. R. P., Varghese, B., Sithambaresan, M., Krishnan, S., Sheeja, S. R. & Suresh, E. (2011). *Polyhedron* **30**, 70–78.

[bb10] Macrae, C. F., Sovago, I., Cottrell, S. J., Galek, P. T. A., McCabe, P., Pidcock, E., Platings, M., Shields, G. P., Stevens, J. S., Towler, M. & Wood, P. A. (2020). *J. Appl. Cryst.* **53**, 226–235. 10.1107/S1600576719014092 PMC6998782 32047413

[bb11] Mahadevi, A. S. & Sastry, G. N. (2016). *Chem. Rev.* **116**, 2775–2825. 10.1021/cr500344e 26840650

[bb12] Olasunkanmi, O., Idris, A. O., Adewole, A. H., Wahab, O. O. & Ebenso, E. E. (2020). *Surf. Interfaces* **21**, 100782.

[bb13] Reena, T. A. & Kurup, M. R. P. (2010). *Spectrochim. Acta A Mol. Biomol. Spectrosc.* **76**, 322–327. 10.1016/j.saa.2010.03.011 20457004

[bb14] Reena, T. A., Seena, E. B. & Prathapachandra Kurup, M. R. (2008). *Polyhedron* **27**, 1825–1831.

[bb15] Rode, K., Maji, I., Mahajan, S. & Singh, P. K. (2024). *Drug Discovery Today* **29**, 104050. 10.1016/j.drudis.2024.104050 38830502

[bb16] Sakhiya, D. C. & Borkhataria, C. H. (2024). *Helion* **10**, e29057. 10.1016/j.heliyon.2024.e29057 PMC11004889 38601657

[bb17] Sheldrick, G. M. (2015*a*). *Acta Cryst.* A**71**, 3–8.

[bb18] Sheldrick, G. M. (2015*b*). *Acta Cryst.* C**71**, 3–8.

[bb19] Siji, V. L., Kumar, M. R. S., Suma, S. & Kurup, M. R. P. (2010*a*). *Spectrochim. Acta A Mol. Biomol. Spectrosc.* **76**, 22–28. 10.1016/j.saa.2010.02.035 20347382

[bb20] Siji, V. L., Sudarsanakumar, M. R. & Suma, S. (2010*b*). *Polyhedron* **29**, 2035–2040.

[bb21] Spackman, P. R., Turner, M. J., McKinnon, J. J., Wolff, S. K., Grimwood, D. J., Jayatilaka, D. & Spackman, M. A. (2021). *J. Appl. Cryst.* **54**, 1006–1011. 10.1107/S1600576721002910 PMC8202033 34188619

[bb22] Taylor, R. & Day, G. M. (2018). *Cryst. Growth Des.* **18**, 892–904. 10.1021/acs.cgd.7b01375 PMC5806084 29445316

[bb23] Westrip, S. P. (2010). *J. Appl. Cryst.* **43**, 920–925.

