# Supplementary material for: Synthesis and structure of a 1:1 1-phenyl­semicarbazide–1-phenyl­pyrazolidin-3-one cocrystal
Source: Acta Crystallogr E Crystallogr Commun. 2026 Mar 17;82(Pt 4):371–4. doi: 10.1107/S2056989026002550 (PMC13055964; doi:10.1107/S2056989026002550)
Supplement: Supplementary file 3 [file e-82-00371-sup4.docx]

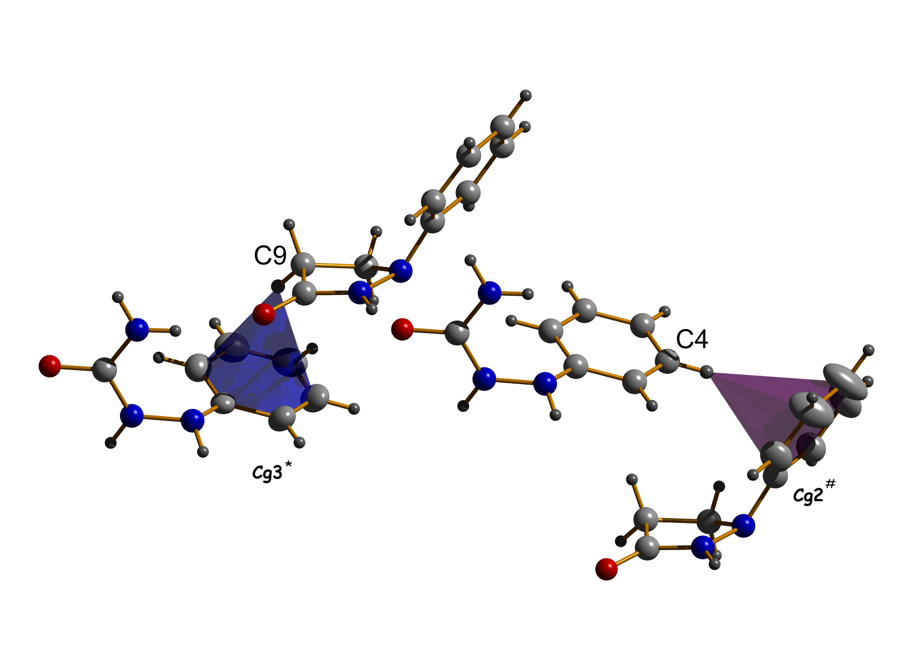


**Fig. S1**. C–H···π interactions in cocrystal ^*^=x,1+y, z; ^#^= 1+_X_, -1+y, z


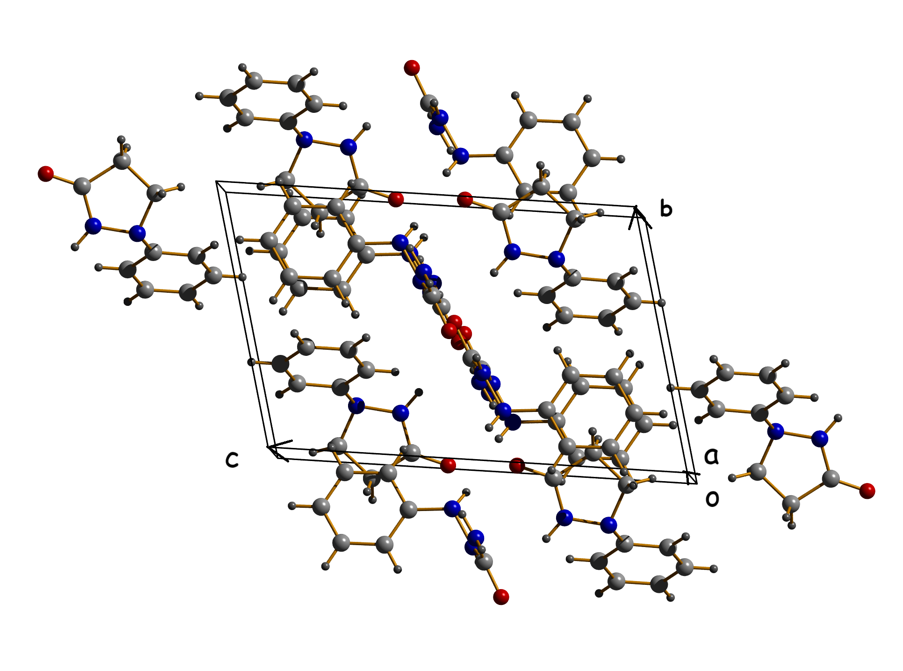


**Fig S2** Packing structure of 1-phenylsemicarbazide-1-phenyl-3-pyrazolidinone. A view down [100] is shown along with the unit cell.


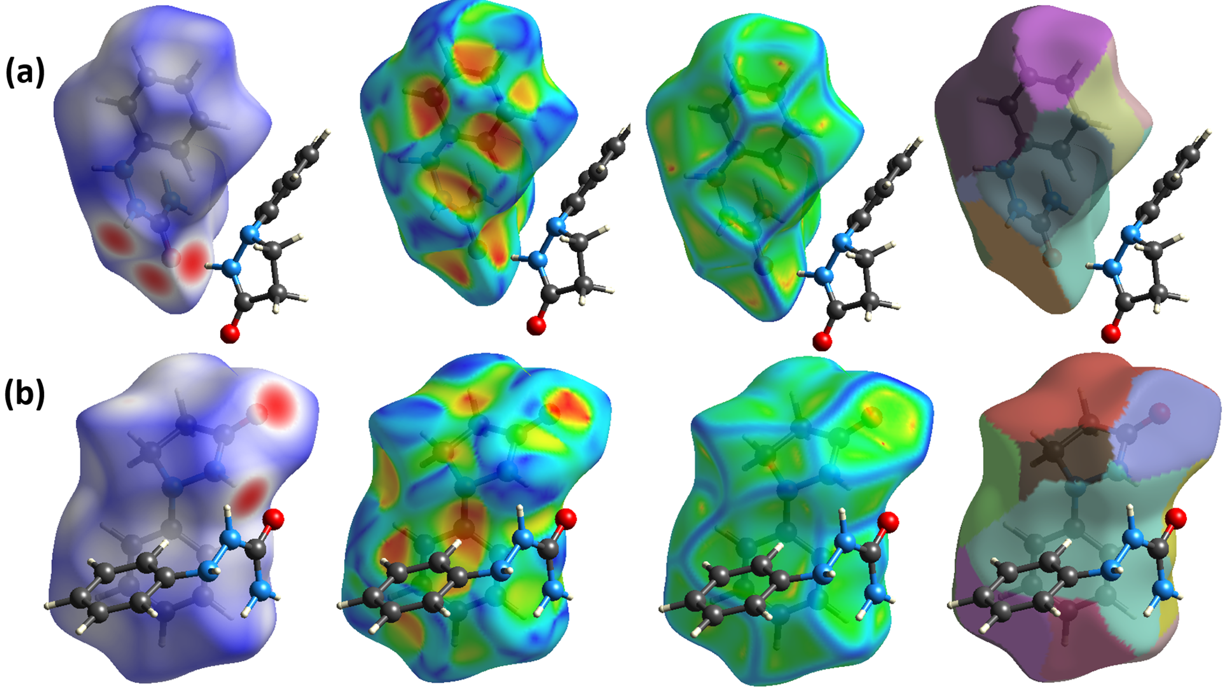


**Fig S3(a) & Fig S3(b)**. Hirshfeld surface analysis for the title cocrystal. The surfaces are mapped over d_norm_, shape index, curvedness, and fragment patches (from left to right) for (a) the 1-phenylsemicarbazide component and (b) the 1-phenyl-3-pyrazolidinone component.


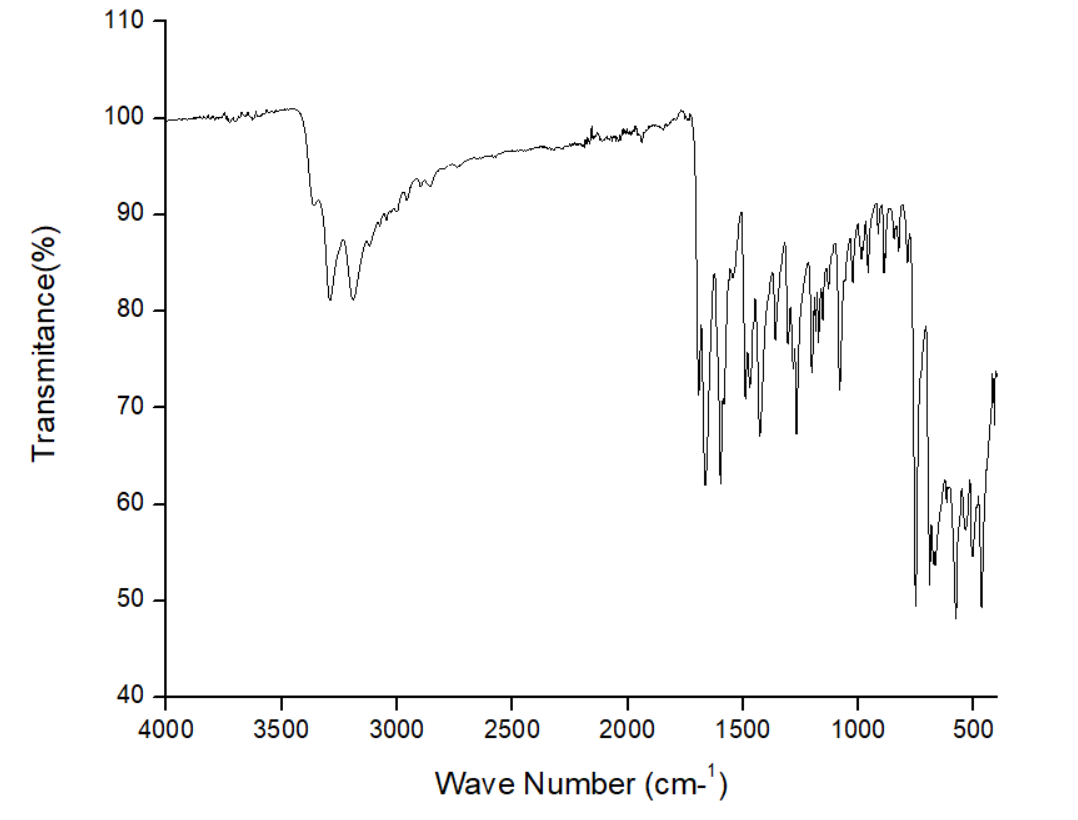


**Fig S4** FT IR spectrum of the cocrystal


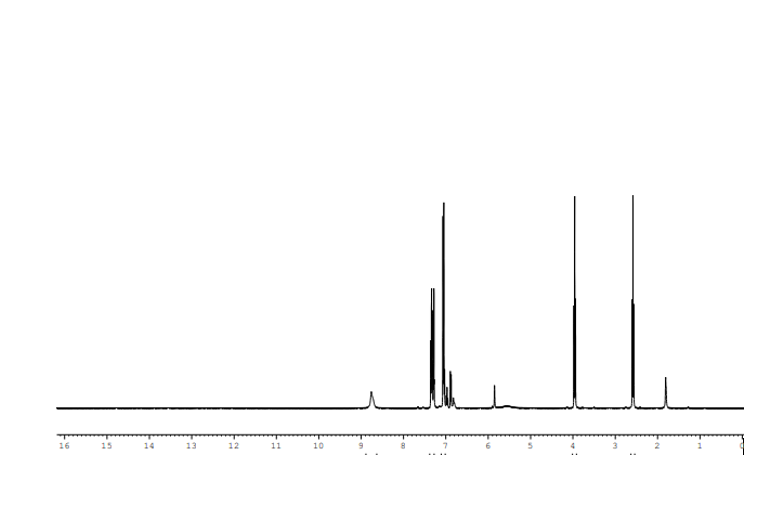


**Fig S5** ^1^H NMR spectrum of cocrystal


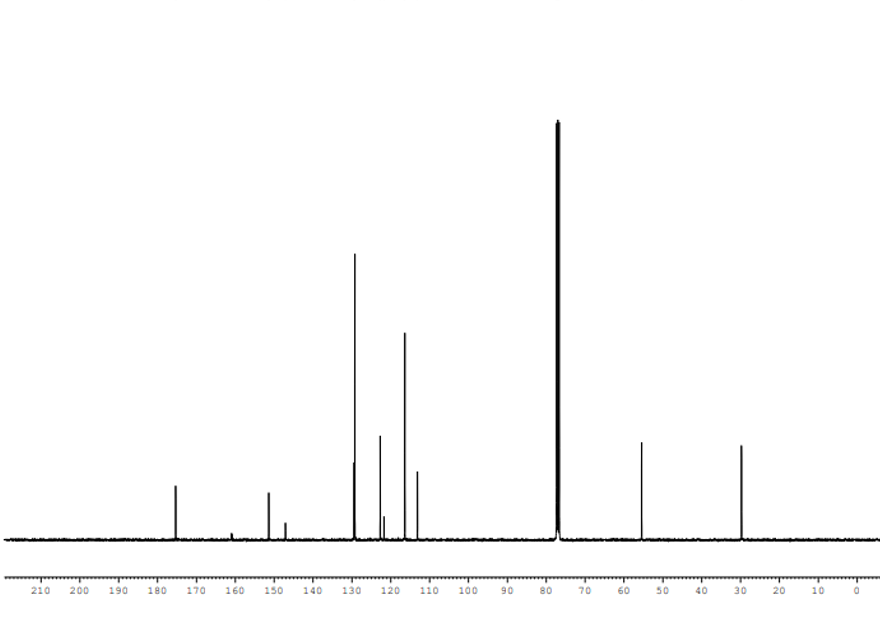


**Fig S6** ^13^ C NMR spectrum of cocrystal


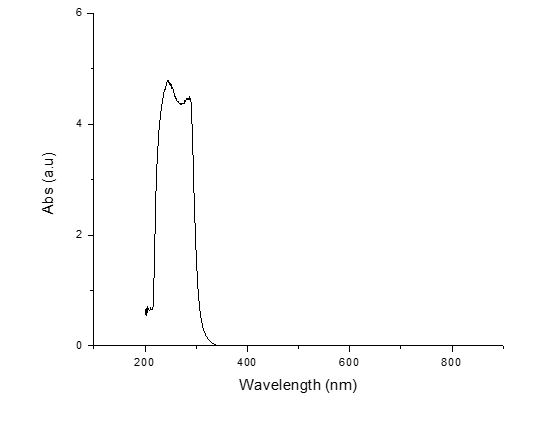


**Fig S7** UV spectrum of the cocrystal
